# The utilization of the concept of profession to understand social problems: sharing preliminary results from systematic review

**DOI:** 10.3389/fsoc.2025.1515427

**Published:** 2025-04-24

**Authors:** Angelique Wildschut, Nomkhosi A. Mbatha, Tamlynne Meyer

**Affiliations:** ^1^Equitable Education and Economies, Human Sciences Research Council, Cape Town, South Africa; ^2^Sociology Department, University of Pretoria, Pretoria, South Africa; ^3^Sociology Department, University of Cape Town, Cape Town, South Africa

**Keywords:** professions, systematic review, conceptual engagement, social problems, sociology

## Abstract

The nature of work has experienced steady shifts that have accelerated over the last three decades, raising important sociological questions; for instance, what does this mean for individuals and groups, and their relation to society, markets and the political systems that contextualize attempts and opportunities for different forms of livelihood? The concept of profession has been a key construct for sociological analysis to understand, study and theorise the implications of such shifts in different countries, workplaces and even particular occupational groups. Studies of professions have thus contributed to knowledge in many ways, not only by highlighting the implications for individuals and groups within work contexts but also illustrating importantly how this relates or not to wider societal phenomena. However, there are strong contentions that because its function as a mechanism of social structure formation has weakened significantly over time, as a sociological category and construct, the concept of profession is no longer relevant in contemporary times. This paper shares preliminary results from a systematic review of literature on the application and conceptualisation of the term profession between 2022 and 2023 to start engaging with the question of whether it has exhausted its sociological relevance. The findings suggest firstly that while there has been an overall decline in the utilization of profession-related terms, a slight increase in the utilization of profession is apparent. Secondly, in the reviewed papers, limited engagement with the conceptual underpinning of the construct exists. Finally, while critical engagement with the concept is not always apparent, most papers recognize a clear link between social phenomena and the role of the profession/s toward maintaining or dismantling such challenges in society.

## 1 Introduction

Historically professions were viewed as important units of sociological analysis, because of their power as societal institutions with the ability to contribute to social order and regulate the power of the market and the state in relation to different social groups (Fournier, 2000). Their role in guiding and governing individuals’ behavior within workplaces as they navigate task delivery have similarly been acknowledged and thus many have argued that professions protect client interests and uphold the quality of services. As Saks (2022, p.2) argues, “they are traditionally seen as an integral aspect of modern neo-liberal societies – as repositories of exceptional expertise, the mainstays of democracy and the guardians of ethical behavior.” Critical to this concept is the idea that individuals belonging to a particular profession have shared identities, culture and value systems constructed around a common knowledge base and set of tasks, practices and rules that govern a particular scope of work.

However, the world of work today is characterized by very different socio-economic parameters, less stable and structured forms of society, much more fragmented forms of livelihood (Ferguson, 2015) and rapidly shifting economic environments, all of which have implications for shaping the future of work, transforming but also transgressing the traditional way work has been controlled, conducted, and conceived (Scully-Russ and Torraco, 2020). Such shifts will continue to challenge and change the notions of what a profession is and what constitutes professionalism, as well as professional values and standards. Additionally, and more importantly, the extent to which these constructs remain relevant to understanding societies and individual behavior in the workplace. It has been argued that because its function as a mechanism of social structure formation has weakened significantly over time, the construct of profession as a sociological category, but also as a strong and significant societal institution, is no longer relevant. As Saks (2022, p. 3) notes, “some academics argue that the focus in modern neo-liberal societies should now be on knowledge and expertise rather than professions themselves, while others believe that professions should be disestablished….”

Rather than merely dismissing professions, a research project was conceived to critically interrogate their role in society, particularly in relation to the perpetuation of inequality. A rich body of knowledge on professions has provided insights into the ways in which social factors shape the distribution of labor and opportunities (see for example, Glucksmann, 2009, 1995; Pareto, 2017; Macdonald, 1995; Freidson, 1988), and how this affects the social structure and dynamics of different societies (see for example, Bataille et al., 2023; McDowell and Court, 1994; Crankshaw, 1996). Thus we argue that dismissing professions as a unit of analysis will occlude clearer understanding of the manifestation, perpetuation and opportunities for dismantling inequalities in society: *at the micro-level within workplaces* between individual professionals or groups of professionals from different segments of society (Meyer, 2018; Meyer, 2024), *at the meso-level where power relations are mediated by the professions in relation to organizations* (Wildschut, 2011; Wildschut and Gouws, 2013; Ashley et al., 2023), and *at the macro-level where powerful professions can challenge government legislation and resist interference when ethically unjust policies or programmes are being proposed* (Saks, 2022; Saks, 2015; Walker, 2005; Bonnin and Ruggunan, 2013).

A small study project, aiming to conduct research that helps us understand professions as societal institutions that have the power to shape, disrupt and reinforce social phenomena at different levels, was initiated. An important starting point is a review of what is known and the gaps in literature that should guide further research, thus we conducted a systematic review of literature. This paper reports on the preliminary findings of this review.

## 2 Method

The team followed a systematic literature review (SLR) approach to engage on the value of profession-related terms for sociological inquiry. This involved a rigorous process of constructing (i) specific research question/s, (ii) literature search, (iii) process of exclusion and inclusion, and (iv) review and elegibility assessment (Xiao and Watson, 2019). While the full systematic review will include four focus keywords (profession, professional, professionalism and professionalisation), the results reported in this paper focuses on the term profession, its usage in the title and abstract, and a period of evaluation spanning 15 months (between 2022 and 2023)^1^. The main aspects of inquiry were whether (i) the papers engaged with the construct of profession, and (ii) whether the research employed the construct to explain a social problem.

### 2.1 Process of investigation

**Figure 1:**
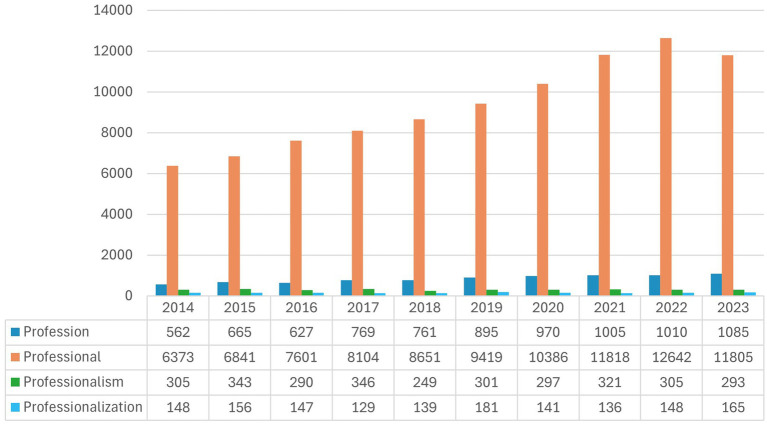
Systematic search results of profession-related terms between 2014 and 2023 (Ebscohost Academic Search Premier, Sabinet and Proquest).

The process of investigation commenced with a systematic search of all profession-related terms (profession, professionalism, professionalization, professional) across the last three decades (Ebscohost Academic Search Premier, Sabinet and Proquest, limited to full text, scholarly journals, the use of the terms in the abstract and title). This already shows some insights in relation to which terms are most prevalent in academic literature, but also the drop between 2022 and 2023 (refer to Figure 1). Although we illustrate only the trend over the last 10-year period in this figure, the high number of results across the 30-year period further supported a smaller initial investigative scope.

This systematic search indicates the magnitude of the task to evaluate changes in the utilization of profession-related terms over the intended 30-year period. To provide insight into how the application of a systematic review methodology could be usefully applied, we start first with a smaller scope of investigation by selecting one term. Thus, to engage more closely, in this paper we focus on the systematic review results of the use of the term profession over a year period (2022–2023), to share emerging insights but also the types of analysis that can be possible as a full project is developed and the dataset improved. The decision to start with a focused analysis of the construct of profession, rather than other related terms, was also informed by established debate over its relevance for sociological analysis (Adams, 2010; Kurtz, 2022) and the role of profession/s in society (Saks, 2022).

### 2.2 Literature identification and screening

Using the keyword ‘profession’, we searched three databases: EBSCOhost Academic Search Premier^2^, Sabinet^3^, and ProQuest^4^ Central. The research criteria comprised peer-reviewed journal papers in all academic disciplines and were confined to the years 2022–2023. Supplementary Table 1 indicates the total number of records that were retrieved from the three different databases.

**Figure 2:**
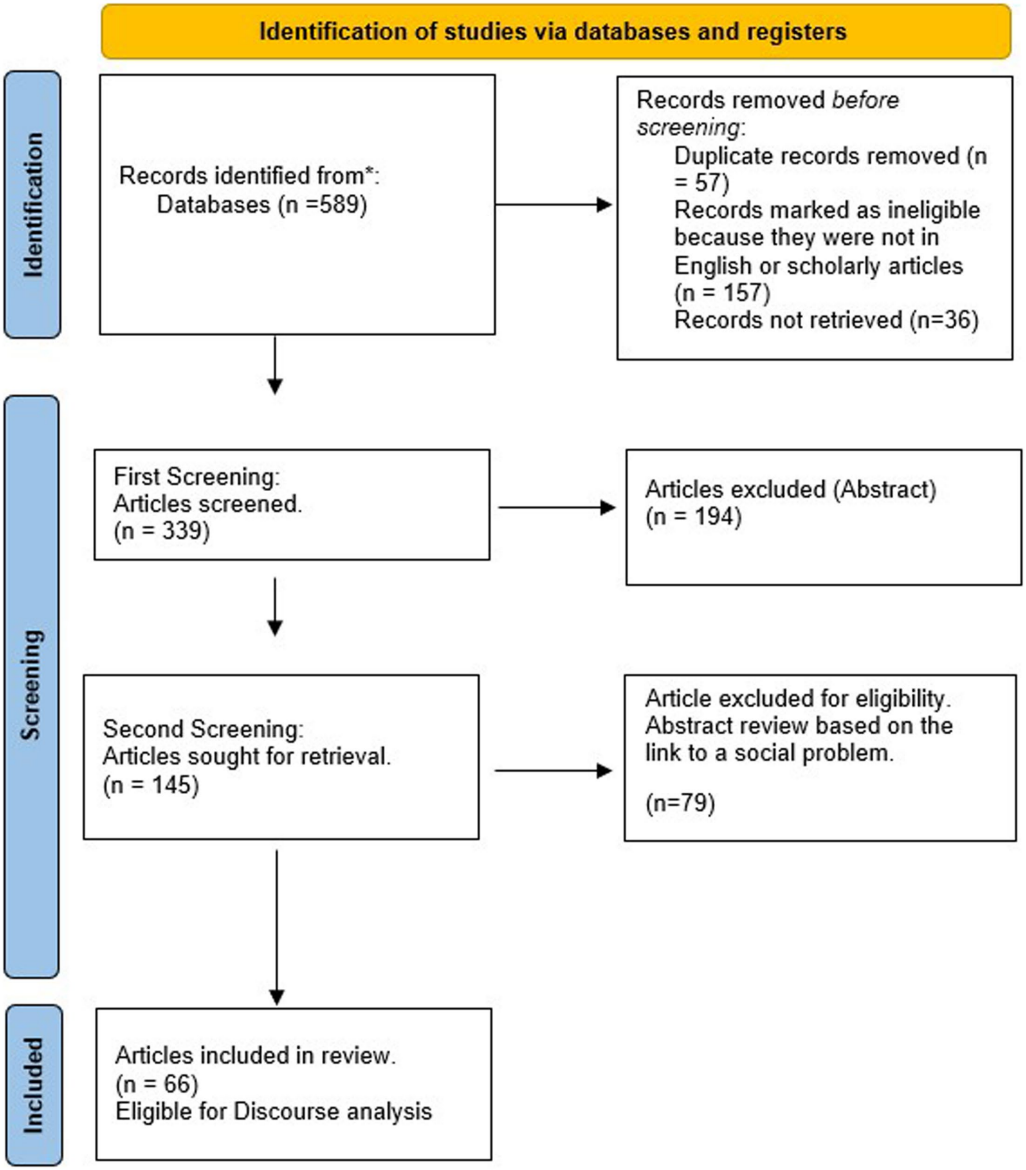
PRISMA* diagram illustrating the identification, screening and inclusion process leading to the final sample of papers. PRISMA stands for Preferred Reporting Items for Systematic reviews and Meta-Analyses (PRISMA) designed to assist systematic reviewers “transparently report why the review was done, what the authors did and what they found” (Page et al., 2021).

Figure 2 illustrates that the literature search resulted in a total of 589 articles being retrieved from the three different databases. In the identification phase, 250 papers were removed as duplicates, non-English and irretrievable. All 339 articles were uploaded into Atlas.ti where three codes were used; (i) country, (ii) profession, and (iii) journal. The country code was used to identify in which country or countries the research was based. The profession code captured which profession/s was covered and finally the journal in which the paper was published. The first stage of the screening process involved the review of abstracts, which led to another 194 papers being excluded based on the review criteria. This screening involved two rounds of review between reviewers. Firstly, there was an assessment as to whether the abstract reasonably suggests that the paper seeks to engage with the construct of profession for analytical purposes. 194 articles were excluded based on an abstract that did not provide insights as to how the article engaged with the construct or how it employed the construct to explain a social problem. This resulted in a total of 145 eligible papers. Secondly, abstracts underwent another round of review, where reviewers excluded papers not explicitly recognizing the link between professions and a social problem (such as inequality, sexism or racism). The remaining 66 papers were selected for full-text retrieval and considered eligible for full review and discourse analysis.

The team engaged on the relevance to sociological analysis by considering two questions. (1) Does the paper engage or interrogate the construct of profession? And (2) Does the paper employ its investigation of profession as a means to understand a social problem?

## 3 Results

### 3.1 Limited conceptual engagement with the construct of profession

Firstly, we found that while the review of abstracts suggested the term profession is used in an analytical manner, most papers do not engage conceptually with the construct at all (40 papers). For the most part, a totally different phenomenon is being studied, and the concept of profession is merely utilized as the context within which it is explored. The journals in which these papers were published can provide some insight into the interpretation of this outcome, examples are, the British Journal of Social Work, New Technology, Work and Employment, Sage Open Nursing, American Journal of Health Professions Education and the American Journal of Clinical Pathology.

Those papers that do engage with the construct fall into two categories. We identified that while some were not attempting to explicitly define the concept directly, they usefully reflect on the sociology of professions literature (Kurtz, 2022; Moreira, 2022; Toraman and Korkmaz, 2022; Llale et al., 2022; Moroșan, 2022) to inform their engagement with a range of topics across various professions – feminisation in the literary profession, professionalism in teaching, the impact of artificial intelligence on quantity surveying and whether the identification of particular criteria is important for considering a field of practice as a profession.

The second category of papers do attempt conceptual discussions, but a few papers still employ the trait approach extensively criticized by the broader sociology of professions field. For example, Lightsey-Tivoli (2022) evaluates the extent to which the instructional technology and distance education (ITDE) field meets the criteria of profession based on Finn’s (1953) definition setting out six criteria: intellectual technique, an application to practical affairs, a defined training period, a professional association including a high-quality level of communication and collaboration, enforced standards and ethics, and expanding theory based on research. Ohmann and Schrecker (2022) similarly apply the trait approach in their discussion of the decline of the academic profession, as does Jehn et al. (2022) in distinguishing between the characteristics of classic and semi-professions, indicating that the latter require less time spent in college, have shorter training requirements and are less exclusive. In a similar fashion, Bolandian-Bafghi et al. (2022) argue that nursing is a profession because of the autonomy, professional commitment, specialty knowledge and skills, shared value system and academic education, whereas Dew and Clark-Grill (2022) indicate a growing appreciation for homeopathy as a profession due to increased societal trust and recognition of indigenous and alternative medicine. Finally, Petrova and Datta (2022) refer to the Standard Occupational Classifications in the United States (US) to define a ‘new occupation’ as one that is not included in the most recent occupational classification system while an ‘emerging occupation’ is one that has small employment numbers but is expected to get larger in the future. We again share some of the journals in which this group of papers were published: Journal of Interprofessional Care, Journal of Law and Society, Canadian Journal of Higher Education, Professions and Professionalism and the Sociology of Health and Illness.

### 3.2 Clear identification of the importance of studying professions to address societal issues

Secondly, we were interested in considering whether the papers employ the construct of profession to understand a social problem. While we have established that there is limited conceptual engagement with the construct, focused analysis shows that more than half of the papers (35) do explore the link between social phenomena and the role of professions.

A substantive number of papers (16) are concerned with the structural inequities brough about by overt and subvert methods of gendering in professions. While there are still some papers concerned merely with the issue of representation (eg. Reimers and April, 2022, which focuses on the architectural profession), predominantly more complex aspects are investigated, mostly centered on the micro-level or implications for professionals within various fields. For example, Hickens et al. (2022) focuses on the impact of ignoring sexual orientation in the dietetic profession and Lică and Negoescu (2017) explores the role of language in perpetuating gender stereotypes and a lowered status for women, while Mumtaz (2022) draws out the implications of gender role conflict, prejudice and office stress for women in two male dominated fields in two countries. Poghosyan et al. (2022) and Durand et al. (2022) examine the gendered effects that might present obstacles to the increasing need for interprofessional teamwork to deliver quality, effective and well-managed care to patients in medicine and nursing. However, there is still a recognition and interest in understanding the impact of gender on profession choice (Canpolat, 2022), with several papers considering particularly the gendered nature of nursing (Shylasree et al., 2022; Shakwane, 2022; Mwetulundila and Indongo, 2022) as well as teaching (Erginer and Saklan, 2022). A related aspect to this discussion is the link between professions and identity formation (Krantz and Fritzèn, 2022; Wagner et al., 2022; Vinson, 2022). For example, Panteli and Urquhart (2022) explore job crafting to showcase how female contractors in IT navigate and achieve well-being and a sense of work/life balance, whereas Prendergast et al. (2022) examine the diversity of pathways to becoming and developing an identity as an assessment professional.

Inequality based on culture and race also emerges as a consistent concern (Ewers et al., 2022; Jefferies et al., 2022) (12 papers touch on this). This appears to be a charge firmly taken up in the papers on the legal field in the form of programmes aimed at improving diversity, equity and inclusion (for, e.g., Razzante and Boss, 2022) and in the medical field in the form of ensuring culturally competent educational programmes and assessment in health professions education (Reicherter and Wruble, 2022), or ‘culturally responsive pedagogy’ (Varnado et al., 2022). A section of papers also deals more generally with the issue of inclusivity. For example, Rasch (2022) considers the role student affairs can play in ensuring a space for neurodiversity in academia, while Reynaert et al. (2022) investigates the practice of human rights through social work. Southerst et al. (2022) examine diversity in the chiropractic profession, whereas Staley et al. (2022) consider the future of speech-language therapy as a profession.

While most papers still tend to be more inward looking, some also consider wider socio-political contexts and the interaction with the institution of profession, as seen in Abrahams et al. (2022) where the links between colonialism and globalization are outlined with implications for students choosing professions aligned to the dominant values and norms of their society. In this collection of papers, clear linkages are drawn between the historical complicity of professions in maintaining inequality but also how addressing social inequality in professional participation holds implications for improved professional practice and more positive impacts in relation to ecological, economic and social contexts. In this regard, Irlam et al. (2023) outline the importance of incorporating a consideration of planetary health and sustainability into the education of health care professions, while Meirkulova and Gelisli (2022) highlight how the social status of teaching in Turkey and Kazakhstan are impacted by regional and country contexts as well as a variety of determinants (excessive workload, incompetence, teacher salaries, economic dissatisfaction and problems with professional development issues).

In line with historical trends, interest in the motivation to enter and status of different professions remains a theme of investigation across a variety of professions (Grinberg and Sela, 2022; Durand et al., 2022; Erginer and Saklan, 2022; Karunakaran, 2022; Kundu and Basu, 2022; Mulder et al., 2022). An emergent theme of investigation reflects a concern with the impact of artificial intelligence on the organization of work. Further investigation reveals that this seems to be an issue of interest, particularly in the financial and accounting related professions, but other professions are also interested in these impacts. For example, Turksoy (2022) examines the impact of AI on public relations, advertising and journalism (7 papers consider these aspects).

There is also a sustained interest in arguing for the recognition of a range of fields of practice as professions and considering shifts in scope (6 papers). This is evidenced in VanSpronsen et al. (2022) focusing on medical laboratory science and Whelan’s (2022) focus on the scope and nature of social work, as well as Wiggins et al. (2022a) examination of the shifting scope of practice of the chiropractic profession and other health care professions Wiggins et al., 2022b. Wright (2022) illustrates how symbolic boundaries are produced in the teaching profession, while Jeong and Kim (2022) consider the impact of BIM on the scope and focus of the architectural profession.

An encouraging insight, as we consider the construct’s relevance to society and the sociological debate on work, is that, although in the main there is limited conceptual engagement with what a profession is, this review of papers has revealed a strong interest in professions. It is interesting that a broad range of journals are represented in these discussions. Summarizing the analysis in terms of topical focus, is equally illuminating for the discussion and opens new avenues for more nuanced analysis.

The continued dominance of research into the medical profession is supported by this review and its preliminary results. However, in addition to the thematic focus in the medical profession still emphasizing issues of representivity in terms of gender and race, the theme of interest on the importance of ensuring diversity, equity and inclusion also emerged as an area of emphasis in papers focusing on the legal profession. While the traditional emphasis on health professions persists, the implications of digitalisation (Innes et al., 2022; Ionescu-Feleagă et al., 2022), the need to address diversity and cultural competence in professions, and the recognition of work change with distinct implications for the scopes of practice of different professions across countries, remains topical for many journals and scholars. In the main, this finding reinforces the social embeddedness of professions.

## 4 Limitation

This paper’s analysis only focuses on the term ‘profession/s’ and does not include the usage of related terms such as professional, professionalisation and professionalism, which has been shown in other works as critical to understand the full discourse around the profession as a social institution (Wildschut and Luescher, 2023). A further limitation relates to analysing papers on the extent to which there is conceptual engagement with the construct of profession and its societal relevance. Of course, not all journals have a sociological orientation, and this would not have been an express objective or aim to adhere to for publication. However, in this regard it is important to not view the analysis as directed toward outlining the limitations of the journals, but rather to assess as comprehensively as possible, the full discourse around the perceived usefulness of professions as an analytical lens for engaging with social problems. In each category of analysis, we anticipated some journals, but were equally surprised with journals we would not have expected to have such extensive conceptual engagement with the construct and its relation to societal phenomena. Relatedly, we anticipate that adding the discipline/field of study of the lead authors of papers would further offer some useful insight to the interpretation of results and will surely be a category of coding we will add to the dataset for future analysis.

## 5 Conclusion

Preliminary results discussed in this paper can be summarized into three overarching points. Firstly, systematic search shows the decline of the usage of profession-related terms recently between 2022 and 2023, alongside a slight increase in the utilization of the term profession. Comparatively, the terms professionalism, profession and professionalization are less frequently used than professional.

Although an initial search suggests that professions remain a clear topic of interest, including in terms of external impacts (skills, knowledge, scopes of practice and identity), closer analysis suggests very limited use of the conceptual tools developed in the sociology of work and professions. This is a clear gap for sociologists to fill by highlighting the social embeddedness of professions and advocating for conceptual engagement with this construct as a social institution shaped by colonialism, globalization, social interaction, lived experiences and multiple positionalities. Disregarding the social embeddedness of professions through failing to engage with sociological theoretical and conceptual frames reduces findings to only empirical descriptions of the nature of various social phenomena but it does not move us forward in addressing these as urgent sites of social exclusion and the reproduction of inequality.

## Footnotes

^1^The period of investigation was influenced by the conference paper presentation in June 2023 in order to cover the most recent literature on professions, the team focused on what had been published a year before inclusive of the first three months of the year.

^2^Academics search premier is the third largest database in the Academic Search family offering access to almost 3,000 active full-text journals, which includes active full-text journals and magazines, active full-text peer reviewed journals, active full text peer reviewed journals with no embargo and active full text journals indexed in the Web of Science or Scopus.

^3^Sabinet provides access to content, technology and research on and from Africa including African Journals, African industry reports, Specialised reference databases and African Archives.

^4^Proquest offers access to dissertations, theses, ebooks, newspapers, periodical and other aggregated databases, but differs slightly from Ebscohost in terms of the disciplinary focus which is more toward business and health sciences as well as also including some trade publications and newspapers from around the world.
