# Supplementary material for: The utilization of the concept of profession to understand social problems: sharing preliminary results from systematic review
Source: Front Sociol. 2025 Apr 24;10:1515427. doi: 10.3389/fsoc.2025.1515427 (PMC12060731; doi:10.3389/fsoc.2025.1515427)
Supplement: Supplementary file 1 [file Data_Sheet_1.pdf]

66 ARTICLES

Number	Author	Title	Mentions	Journal	Does it engage with the concept of profession?	Does it employ the construct as a means to understanding a social problem?
**1.**	Abrahams et al.	Emerging professional identity formation: Exploring coloniality in the rehabilitation professions.	25	*Professions and Professionalism*	**Yes**, the article explores the key drivers towards professional identity formation especially amongst undergraduate students. The article further explores the social embeddedness on professional identity formation and how the categories of identities when applying intersectionality help students to apply meaning to their professional identity.	**Yes**, especially in recognizing the differences between intersectional identities and professional identities. Moreover, social embeddedness e.g., colonialism or globalization are some of the factors that shape professional identities of students. Students ascribe to the dominant values and norms of society that act as key drivers when choosing a profession particularly that will be aligned to such
**2.**	Almufadda et al.	Artificial intelligence applications in the auditing profession: A literature review.	22	Journal of Emerging Technologies in Accounting	**Yes**, the authors are exploring negative and positive impact of artificial intelligence (AI) towards the efficiency, effectiveness, and quality of work of accounting professionals. The article argues that while there is no profession that is immune from the impact of AI especially when they can do certain tasks like humans, the professional path of accountants is being transformed. The nature of work of accountants is being redefined for them to engage in different roles where they can acquire more skills.	**Yes**, the article engages with the accounting 'professional' construct to explain how the profession is being changed by AI. While using the term 'profession' the authors highlight that accounting or auditing is not dying rather offer a suggestion that this is a profession that will thrive alongside AI. Although AI may replace some parts of the work that are automated the profession itself is forced to focus on helping both accountants and auditors to improve their career paths by integrating AI systems into their and assume more of an advisory role. In essence the article highlights the positive impact of AI within the accounting profession.
**3.**	Bolandian-Bafghil et al.	Nurses' experiences of the role of organisational and environmental factors in the development of love of the profession. A qualitative study.	79	*BMC Nursing*	**Yes**, this article engages with the construct in order to explain the concept 'love of profession' (LOP) within nursing. LOP is described by the author as based on values such as love and the ability to care for others	**Yes,** the authors engage with LOP in the article in order to articulate the significant factors for LOP being family support, organizational environment, and health. Both of the above factors ae instrumental for the social status of nursing and the degree to which LOP increases among nurses.
**4.**	Caldararo	Destroying institutions: Abandoning the medical	8	*Journal of International Relations*	**No**, this article discusses the how institutions are affected by the attitudes of populations. Therefore, highlights the difficulties faced by	**No**, the article merely discusses the role of institutions in society and the difficulties faced by medical institutions. The author does not engage with the term 'profession' to describe how medical practitioners in particular may have

		profession during a pandemic.			medical institutions during Covid-19 as they came under attack facing multiple lawsuits.	been impacted and how institutions protected or subjected them to the judgement of the public.
**5.**	Caldis	'Overwhelmed' and 'Underprepared': The realities of out-of-field teaching in Geography during a time of transition into the teaching profession.	23	*Geography Bulletin*	**Yes**, the article explores the experiences of teachers who have just entered the profession facing 'out-of-field-teaching (OOFT). OOF is a phenomenon within the teaching profession where teachers teach subjects that they are not trained to teach. This article explores how early career teachers are affected and how can they be supported.	**Yes**, to broadly explain how in the teaching profession there are challenges faced by those that are transitioning into the profession. In a way this author explains how OOFT can be addressed by the experts within the profession to address the difficulties faced by early career teachers and also to ensure effectiveness and efficiency within the profession.
**6.**	Canpolat	The most feminine and masculine professions according to university students: A mixed methods	6	*International Online Journal of Educational Sciences*	**Yes**, this article identifies how gender constructs influence how students choose professions. Also, how such constructs influence student perceptions about certain departments in universities either being masculine or feminine.	**Yes**, especially in discussion how the gender construct has a role in defining and highlighting the job opportunities that must be pursued by students.
**7.**	Coman et al.	Challenges of the accounting profession in the digital era: The impact of cloud computing technology.	9	*Annales Universitatis Apulensis Series Oeconomica*	**No**, this is an article about awareness of digitization affecting the accounting profession and how accountants must adjust.	**No**, this article is explaining the important of cloud computing storage and technological shifts within the accounting profession. It is a recommendation for the accounting professions to explore the consequences of digital technologies and the modernization of accounting.
**8.**	Constantin et al.	Digital entrepreneurship via sustainable online communication of dentistry profession, Oradea,	10	*Sustainability*	**Yes**, the article explores how dentistry as a profession has an entrepreneurial orientation towards its. The authors analysis describes the roles of social media advertising in the success of dental professionals in Romania.	**Yes**, the authors explain professional specificity and business sustainability in dental profession. They further explain how sustainability is important in the profession including factors such as economic, social, and environmental.

		Romania: A longitudinal analysis.				
**9.**	Dew et al.	Routes into homeopathic profession: Witnessing, gender and subaltern therapeutics.	30	*Sociology of Health and Illness*	**Yes**, as much as homeopath is a profession that is attacked by those that hold traditional beliefs about medicine (classical profession) and do not believe in alternative medicine. This article describes the factors that make people get drawn to homeopath as a profession for clinical practice.	**Yes**, the article engages with the term 'profession' to highlight first why homeopath in its own right is a profession within the medicine discipline although it falls in the category of alternative medicine. Moreover, since it involves clinical practice why specifically people choose it for healing rather than traditional medicine and those who practice it why are they drawn to it.
**10.**	Dooley	Establishing a profession through boundary drawing: Defining criminology's autonomy vis-à-vis six competing disciplines.	4	*American Journal of Criminal Justices*	**No**, this is a critique about criminology and an evaluation as to whether it is an independent profession or is it dependent to other professions or disciplines such as sociology.	**No**, while the author explains what criminology is, the critique seeks to illuminate that criminology cannot exist solely as a discipline and it can't separate itself from other disciplines that provide a foundation for it.
**11.**	Durand et al.	The role of gender, profession and informational role self-efficacy in physician-nurse knowledge sharing and decision-making.	40	*Journal of Interprofessional Care*	**Yes**, this article describes the relationship between gender and professional status. The argument presented by the author is that the societal gender construct has an influence on professional status. Especially in the professional relationships between healthcare professionals affects self-efficacy which are the capabilities that one possesses to succeed in their profession and critical for job satisfaction.	**Yes**, the authors engage with the 'profession' construct to explain how gender affects informational role self-efficacy (IRSE) for medical professions which is a critical skill. This article addressed the gap in medical professions by exploring this phenomenon because female employees tend to struggle with IRSE as they deemed not knowledgeable enough to make decisions or share information with other profession in relation to the wellbeing of patients.
**12.**	Erginer et al.	The problem of transformation of the teaching profession into a female profession: Views of the	18	*HU Journal of Education*	**Yes**, the authors engage with the teaching profession not only as a job but as job that is suitable for which gender in society. The article therefore explores the perceptions and the status of this profession in Turkey and why students consider it or what factors pull or push them towards the teaching profession.	**Yes**, to explain the push and pull factors towards the teaching profession and to explain why society perceives the profession as a calling for women rather than men.

		prospective teachers.				
**13.**	Ewers et al.	"Oh, you're my health care provider?" Recounting the experiences of people of African descent in Nova Scotia pursuing or working in health professions.	18	*CMAJ*	**Yes**, the paper highlights the challenges faced by African diaspora that is pursuing or is working in the health professions.	**Yes**, in order to explain the challenges faced by African diaspora. Also highlight the strategies they engage in to navigate the challenge they face as they enter the health care profession.
**14.**	Gonzalez-Mendoza et al.	University professor: A profession with diverse worlds.	2	*Journal of Language and Linguistic Studies*	**No**, this is an analysis on the performance, knowledge production and administrative duties of Colombian professors.	**No**, the authors focus on how professors focus on both academic and administrative duties. The paper specifically focuses on the professor's scientific performance by looking at research, interactions with society, and knowledge production.
**15.**	Grinberg et al.	Perception of the image of the nursing profession and its relationship with quality care.	38	*BMC Nursing*	**Yes**, this article examines the relationship between self-image and the nursing profession.	**Yes**, there are many factors that are affecting the image of the nursing profession. This article engages with the construct in order to address issues that surround the image of the nursing profession. At the same time how, positive image of the nursing profession can be created especially exploring the role of policies, societal perceptions, and empowering nurses since all the above are critical in nursing care.
**16.**	Hickens et al.	"It literally can save lives": How challenging structural inequities in sexual orientation and gender priorities can create change in the Canadian	31	*Canadian Journal of Dietetic Practice and Research*	**No**, this is an article that explores the provision of dietetics services to and how sexuality inequalities can be addressed.	**Yes**, the author engages with construct to help understand how the LGBT community has faced inequities and how the dieticians in the services they offer can be able to address those inequities.

		dietetic profession.				
**17.**	Holmes et al.	Artificial intelligence: Reshaping accounting profession and the disruption to accounting education.	41	*Journal of Emerging Technologies in Accounting*	**Yes**, this article provides analysis on how accounting professionals view AI in relation to how they can benefit from it as far as business is concerned.	**Yes**, the authors are looking to into the future of work of the accounting professionals and the integration of robotics. The article helps to understand how robotics and digitization ought to be central for the education of accounting professionals.
**18.**	Inness et al.	Social representation of psychology and the application of artificial intelligence: European Union regulatory authority and the application of psychology as a paradigm for the future.	30	*Australian and New Zealand Journal of European Studies*	**Yes**, this is an article about understanding how AI can transform psychology as a profession and how the EU is governing the digitization process within the profession. The central argument on this paper is that while AI might have serious implications in the practice of psychology. There are however tasks that it can help with such as algorithms that are used in evaluations. Because of the serious consequences that it bears it is important that the governance of AI in the discipline not only controls how it is applied but its development and the tasks it will perform are formulated within the profession.	**Yes**, in order to understand psychology not as a discipline but as profession and its relationship with AI. Psychology as a profession has responsibilities over people where the role of a computer should be monitored and limited.
**19.**	Ionescu-Felaga et al.	Empirical evidence on the development and digitization of the accounting and finance profession in Europe.	112	*Electronics*	**Yes**, this is an article that evaluates the development and digitization of services offered by accounting professionals. The author further looks at the role played by the accounting professionals regulation bodies in governing how technology is used by accounting professionals.	**Yes**, technology has become critical in the accounting profession. It is imperative to understand how this profession is governed and regulated when it comes to the use of technology and in maintaining standards when it comes to client needs. Therefore, the author engaged with the construct to understand technology in relation to accounting as a profession.
**20.**	Irlam et al.	Planetary health and environmental sustainability in African health	12	*African Journal of Primary Health Care*	**No**, this is a call for the integration of planetary health and sustainability in the education of Health Care professionals.	**No**, this call for participating in transforming Africa's Health professions curricula by integrating planetary health and sustainability. Also. To recognize the role of healthcare professionals in meeting the SDGs.

		professions education.		*& Family Medicine*		
**21.**	Jeffries et al.	Black nurses in the nursing profession in Canada: A scoping review.	55	*International Journal for Equity in Health*	**Yes**, this study unpacks the history of the nursing profession in Canada and how Black nurses have been positioned within the profession. The authors pay attention on how structural and institutional factors have shaped the profession when it comes to issues of diversity and racism.	**Yes**, by applying the term 'nursing profession' the authors are investigating the intersectional identities of nurses within the profession. Also, how the profession has struggled over the years by being restrictive towards the practice and training of Black Nurses. The nursing profession as an institution, needs to engage and address issues of diversity especially when comes to acknowledging the clinical and practice contributions of Black nurses.
**22.**	Jehn et al.	Training and employment of classic and semi-professions: Intensifying versus accommodating logics.	149	*Canadian Journal of Higher Education*	**Yes, t**his is an article that focus on classic and semi-professions. The authors make a contrast between these two types of professions. Classic professions that usually perceived as prestigious professions are those that require 'complicated' and esoteric knowledge, exclusive and training programs long such as Legal, Pharmacy, Doctors. Semi-professions that require less time spent at college like Social Work and Teaching have shorter training requirements and are less exclusive.	**Yes**, the discussion of the authors in this article not only highlights the major differences into these professions however draw on the fact that the labour market outcomes shape these differences because high wages earned by classic professions sets them apart from the semi-professionals. Therefore, the construct is applied to explain the accommodating logic that differentiates classical professions and semi-professions.
**23.**	Jensen et al.	Changing cultural conditions for knowledge sharing in the teaching profession: A theoretical reinterpretation of findings across three research projects.	65	*Professions & Professionalism*	**No**, the article is about professionalism rather than professions. It investigates the significance of knowledge sharing in development practices and in maintaining professionalism. Within the teaching profession the article looks at how teachers define professionalism within the profession and how such practices are regulated defined when it comes to knowledge sharing.	**Yes**, the article applies the construct to help understand what professionalism is in the context of teaching profession. To understand how within the 'profession' knowledge sharing practices are applied and relate to culture and profession context. The context of the profession is what the authors highlight in this article to unpack how professionalism practices emerge as way of facilitating knowledge sharing therefore, triggering epistemic work.
**24.**	Karunakaran	Status-authority asymmetry between professions: The case of 911	20	*Administrative Science Quarterly*	**Yes**, the author is looking at the role of professionals within the workplace and examines how status asymmetries between professions of low rank and high rank emerge.	**Yes**, to explain and unpack the asymmetries that exist between professions. These asymmetries maybe caused by gendered, status professions or caused by workplace processes and structures.

		dispatchers and police officers.				
**25.**	Krantz et al.	Changes in the identity of the teaching profession: A study of a teacher union in Sweden from 1990 to 2017.	36	*Journal of Educational Change*	**Yes**, this is an article that focuses on inequalities among teachers at school and the role of the trade unions within the teaching profession. The authors investigate the formation of collective identities at the same time engage in a CDA to evaluate the different representations of collective identities.	**Yes**, even though there is a discussion about social practice that trade unions need to engage with in order for them to address the challenges faced by teachers. In applying the CDA the article highlights the link that exists between trade unions and the teaching profession. Therefore, the construct is applied to help understand the collective identities of teachers and the role of trade unions within the profession.
**26.**	Kundu et al.	Latent feminization in teaching profession: Questioning patriarchal hegemony in Greater Kolkata, India.	61	*IASSI Quarterly: Contributions to Indian Social Science*	**Yes**, the article investigates the factors propelling young females to pursue the teaching profession as a career path. The author argues that the feminization of the teaching profession while related to the increasing numbers of women joining the profession is also related to the 'maternal impulses' that society expects women to ascribe to.	**Yes**, the authors engage with the construct in order to explain why there is an increase of women within the teaching profession in India and the numbers of men are declining. The authors explain use the profession to highlight the gender disparities in Indian communities and how teaching is supposedly the right profession for women because the identities that they possess and duties to fulfill.
**27.**	Kunkel	Artificial intelligence, automation, and proletarianization of the legal profession.	42	*Creighton Law Review*	**No**, this article is a about the practice of the legal profession and the incorporation of AI systems. The article interrogates how not only technological systems will have an impact in the future of work of legal practice.	**No**, there is a discussion about the impact of AI in practice and why law should be able to adapt to the technological advances.
**28.**	Kurtz	The end of the profession as a sociological category? Systems-theoretical remark on the relationship between profession and society.	126	*The American Sociologist.*	**Yes**, this is a sociological analysis of how profession together with the terms professionalism and professional are applied in everyday language. The article provides a theoretical overview of the definition of the term and how the classical theorists of sociology have used the term to describe or link it to the way society functions.	**Yes**, this article does not use the construct to explain a social problem but engages with the construct as a sociological concept in relation to social theory. Therefore, there is a discussion on how professions are related or shaped by society and how especially classical professions are being transformed as society is getting modernized.

**29.**	Lalle et al.	Experts' perspectives on the future of the quantity surveying profession.	57	*The International Journal of Technology, Knowledge and Society*	**Yes**, the article explores the quantity surveying profession and how quickly it is changing especially being affected by automation. Furthermore, there is an investigation on how this 'profession' can continue to thrive and retain its status as one of the classical professions in the future.	**Yes**, to understand the predicament faced by the 'profession'. The author engages with the construct to explain future of work for those that are in the quantity surveying profession and the precarities of the job.
**30.**	Lightssey-Tivoli	Characteristics of a profession.	18	*Distance Learning*	**Yes**, evaluates the extent to which the instructional technology and distance education (ITDE) field meets the criteria of profession based on Fin's (1953) definition setting out 6 criteria: intellectual technique, an application to practical affairs, a defined training period, a professional association including a high-quality level of communication and collaboration, enforced standards and ethics, and expanding theory based on research.	**No**, there is no critique in using Fin's definition to examine the extent to which the field can be characterized as a profession. There is also no engagement with how the construct sheds light on a social phenomenon.
**31.**	Marcotrigiano et al.	The Preventive Health Professions in Italy: The Efficient Use of Resources, Skills and Best Practice during the Pandemic.	2	*Healthcare*	**No, t**he focus of the paper is to assess how two categories of health care workers (health visitors and environmental health officers in Italy) dealt with the required reallocation/task shifting of certain aspects of their scopes during COVID 19 and how this aligned with their perceived levels of fatigue and pressure and the extent to which they felt that their training and skills prepared them to take on these aspects successfully.	**No**, it is 'inward' looking as it evaluates the impact on skills and education requirements deriving from an external driver (COVID 19) and links this to training requirements for such professionals within health system especially during times of rapid change and challenge.
**32.**	Meirkulova et al.	The Social Status of the Teaching Profession: Teachers' Views in Turkey and Kazakhstan	90	*Hacettepe University Journal of Education*	**No**, the paper examines the perceptions of Turkish and Kazakh teachers in relation to the social status of the profession. The research highlights different aspects of the regional and country context that impacts on teacher's social status (excessive workload, incompetence, teacher salaries, economic dissatisfaction and problems with professional development) and closes by suggesting ways to elevate the status drawing from the identified issues. An important recommendation is for the enactment of the Teaching Profession Law which would clarify,	**No**, it is 'inward' looking as it evaluates how professionals view their profession and the aspects that lead to low status. It does not use profession as a unit of analysis to explore or understand a wider social phenomenon.

					improve and protect the employee rights of teachers in the educational system.	
**33.**	Michaila Lica et al.	Considerations regarding the gender of professions in the Romanian Language	1	*Land Force Academy Review*	**No**, but does engage with how language can contribute to the continued gendering of professions. Even in attempts to feminize for political correctness, the result can still be derogatory or unequal treatment or assignment of status to women within professions. The author cautions thus that as languages change one needs to be careful to not change in such a way that is unnatural which reinforces inequalities rather than disrupts it.	**Yes**, the article looks at how different languages employ masculine and feminine prefixes or versions of the title assigned to a woman and a man in the same profession and how these can perpetuate gendered stereotypes and a lowered status for women.
**34.**	Mitchell et al.	Clinical trial management: a profession in crisis?	5	*BMC Trials*	**No**, engages with the issues for attracting and retaining clinical trial managers. The paper outlines the critical role these professionals play in meeting the national health plans of a country and argue that the value and highly specialized nature of this role should be promoted.	**No**, it is 'inward' looking – evaluating the complexity of issues that need to be addressed to retain and attract clinical trial managers within the UK system.
**35.**	Moreira	Arbitration vis-à-vis other professions: A sociology of professions account of international commercial arbitrators.	34	*Journal of Law and Society*	**Yes**, details insights from the sociology of professions and how different phases of theorization has impacted on the arguments around the characteristics that define a profession and whether it is important to try to create a list of aspects.	**No,** but it does describe the peculiarities of international commercial arbitrators to the extent that it highlights some aspects of market closure that will result in unequal prospects for access to new entrants, while there does not appear to be formal closure mechanism supported by the field.
**36.**	Morosan	Romanian women writers and the literary profession during the first half of the 20th century: Exclusion, feminisation and the professionalization of writing.	26	*Metacritic Journal for Comparative Studies and Theory*	**Yes**, a historical consideration of the feminization of the literary profession in Romania and draws from different conceptualizations of what is required in a profession to debate the positives and negatives of the feminization process, using constructs from Bourdieu.	**Yes**, through detailing the different phases of the feminization of the literary profession in Romania over time shows how symbolic capital of masculine elites can be reproduced through different aspects, institutions and discourses surrounding the inclusion of women in the profession.
**37.**	Mosch et al.	The medical profession transformed by	11	*Digital Health*	**No**, considers the implication of AI for the scope and nature of the medical profession.	**No**, it is 'inward' looking – considering the changes to the job profile and professional role of physicians to new forms

		artificial intelligence: Qualitative study.				of human AI collaboration and shifts to higher value activities
**38.**	Mulder et al.	Influence of social networks in healthcare on preparation for selection procedures of health professions education: A Dutch interview study.	1	*BMJ Open*	**No**, considers the nature and role of social networks in influences motivation to enter into HPE	**No**, although it is exploring how social networks facilitate and shape entry and decision to enter into the medical profession and this results in unequal access to the medical profession which has implications for the inclusivity and representativity in the profession of people from 'non-traditional' backgrounds.
**39.**	Mumtaz	Does gender differences matter! Investigating the prejudiced practices and gender role conflict among female professionals working in male-dominated professions.	3	*International Journal of Business and Society*	**No**, explores the relationship between gender role conflict, prejudice and offices stress of women in two male dominated fields in two countries: Malaysia and Pakistan.	**Yes**, through exploring direct and indirect and moderating relationships between discriminatory acts, emotional and psychological imbalances, work-family conflict and household responsibilities and financial needs across two countries the differences in how women experience these aspects and the implication for their progression through these field was illuminated. The analysis is quantitative and positivist however.
**40.**	Mwetulundila et al.	Gender Equity and Impedes of Career Progression in the Nursing Profession in Khomas Region of Namibia.	7	*SAGE Open Nursing*	**No**, does not define profession. Explores gendered distribution in the nursing profession.	**Yes, t**hrough exploring the qualification profiles and distribution of women and men in the nursing profession identifies structural impediments to advancement that are organizational and not necessarily gendered. However it does confirm the importance of higher levels of qualification for advancement and that women are less likely to pursue these as there are other benefits of being at a lower rank (e.g. Overtime).
**41.**	Ohman et al.	The decline of the professions.	8	*Radical Teacher*	**No**, applies the trait approach to underpinning the discussion on the decline of the profession.	**No**, inward looking in that it details through a somewhat historical overview what broader macro and political changes have impacted on the decline of the academic profession particularly but professions in general as well.
**42.**	Panteli et al.	Job crafting for female contractors in a male-dominated profession.	20	*New Technology, Work and Employment*	**No**, focuses on the phenomenon of job crafting and explores it within the context of female contractors within the IT profession. It is grounded in the literature on contracting as an increasingly new form of non-traditional workers	**No**, however, in exploring the flexibility and autonomy that women craft through engaging in contracting work within the IT profession, shows was in which they can progress and find levels of balance that works for them and their sense of wellbeing and work life balance. It attempts to address the

					that have boundaryless careers with physical and psychological mobility.	absence of gender in mainstream organizational research that leads to a notion of an 'ideal type' worker who has no family or caring responsibilities. The study highlights the gendered nature of permanent IT work and through moving into contracting was a way for these women to take control of their work experiences.
**43.**	Petrova et al.	Value sustainability of emerging social commerce professions: An exploratory study.	31	*Information*	**Yes**, to some degree in reflecting on the emergence of these new professions refers to recognized profession classifications in the US's SOC which mentioned 'blogger' as an addition to the category of writers and authors. It refers to a new occupation as one that is not included in the most current occupational classification system while an emerging occupation is one that has small employment numbers but is expected to get larger in the future.	**Yes**, focuses on value creation and co-creation processes in the development and delivery of new professions that can add to the social capital of the society by creating sustainable value. The focus is thus on value creation processes and social interaction flows and the research evaluates this in the context of new and emergent professions (vlogging and bogging) through leveraging mobile technology and social media. However, this is not grounded in the SoP literature but the value creation and co-creation and user acceptance of technology literature.
**44.**	Poghosyan et al.	Physician and nurse practitioner teamwork and job satisfaction: Gender and profession.	22	*The Journal for Nurse Practitioners*	**No**, focuses on gendered effects in nursing and medicine with a view towards understanding the links between job satisfaction and professional identity. The aim is to understand gendered effects that might present obstacles to the increasing need for interprofessional teamwork to deliver quality, effective and well-managed care to patients.	**No**, inward looking in that the focus is on gendered effects across and within the two professions but the implications for quality care is not discussed or analysed comprehensively. The findings are however useful in highlighting the different role of gender across the two professions. Profession in this study, moderated the relationship between gender and team and gender and job satisfaction.
**45.**	Prendergast et al.	Fields, Professions, and Disciplines: Exploring Professional Identity in Assessment.	14	*Research and Practice in Assessment*	**No**, examines the diversity of pathways assessment practitioners take into becoming professionals and the differences in conceptualization of assessment work as profession, field and specialization.	**No**, does touch on some of the skills and knowledge requirements of assessment work and how this has changes to be more holistic and student centred as opposed to the past where it was more teaching centred. It highlights the role assessment practitioners can play in the professional development of the field towards the future and also how they can play a role in ensuring equality within the delivery and assessment of learning.
**46.**	Rasch	The lights are too loud: Neurodivergence in the student affairs profession.	10	*The Vermont connection*	**No**, the focus is on neurodivergence within the higher education environment and focuses on the role of the student affairs profession can play to facilitate inclusivity.	**Yes**, while not employing sociological constructs the paper uses profession as a way to evaluate the role that professionals can play in ensuring greater inclusivity within higher education. The paper seeks to amplify neurodiverse students and student affairs professional's experiences and a way to offer recommendations on how diverse voices can be used to deconstruct inequitable systems.

**47.**	Razzante et al.	DEI in the legal profession: Identifying foundational factors for meaningful change.	22	*Utah Law Review*	**No**, engages with diversity, equity and inclusion in the legal profession through power, intersectional and other theories.	**Yes**, the essay explores the complexity of identity and power relations and how the classroom and workplace environment can be used to create and support opportunities for acknowledgement of diversity, equity and inclusion. Not only does the essay explore these themes as it relates to the inclusion of marginalized social groups within the profession, it also touches on the notions of equity as it is espoused in the law and which actually requires critical reflection in classroom and work spaces to enable true change and transformation.
**48.**	Reicherter et al.	Health Professions Program Evaluation: A culturally competent and responsive framework.	0	*Journal of Allied Health*	**No**, applies the construct of profession without interrogating what it means. A focus on health professions education. No. Focuses on HPE programs and ways to ensure culturally competent educational programmes and assessment.	**No**, engages with social problems and the role that HPE plays in perpetuating or the opportunities it has to disrupt it, but does not focus on the profession as a means to do so. No. Focuses on ways in which HPE programmes can become more culturally competent both in its delivery and assessment in such a way that can foster diversity in the teaching and learning and educational experience but also through including and ensuring the success of a more culturally diverse student population has the potential to ensure more culturally competent and a more reflective health profession. The argument is that creating a highly skilled and diverse workforce capable of decreasing health disparities arising from ongoing social injustices, health professions programs must ensure the cultural responsiveness of all aspects of their student's educational journey.
**49.**	Reimers et al.	An intersectional approach beyond race and gender: Women in the SA Architectural Profession.	63	*Effective Executive*	**No**, explores women's representation in the architectural profession.	**No**, uses intersectionality as theoretical frame wot explore the position of women in architecture and does not reflect on architecture as a profession and the role it plays explicitly. Foreground the individual experience and thus the focus is more on lived experience, there is some linkages made to culture in the profession, conscious and unconscious bias, gender role reinforcement and attrition but not from a sociological standpoint.
**50.**	Reynaert et al.	Social Work as a Human Rights Profession: An action framework.	15	*British Journal of Social Work*	**No**, explores the practice of human rights through social work recognized as a human rights profession.	**No**, proposes an action framework for human rights in social work, but acknowledges the constructed nature of human rights within the field. The paper contributes by proposing five building blocks as an action framework for human rights in social work: system world orientation,

						lifeworld-oriented action, participatory action, joined-up action and politicizing action.
**51.**	Shakwane	Men in service of humanity: Sociocultural perceptions of the nursing profession in South Africa.	16	*African Journal of Health Profession Education*	**No**, the concept of profession is applied without critique and the paper explores the gendered nature of the nursing profession from the perspective of male nurses.	**No**, does not interrogate deeply the way in which the gendered notions are inculcated in the profession. The findings offer some counterpoint to the general tendency in the literature to highlight the privileged role that men tend to play in the nursing profession, highlighting cultural and social context factors that might play a particular role in the discriminatory context that male nurses in the South African context face.
**52.**	Shylasree et al.	Maternity and childcare domain of women in medical profession.	4	*Indian Journal of Medical and Pediatric Oncology*	**No**, the concept of profession is not interrogated or explained.	**No**, the paper is 'inward looking' and merely a descriptive account of the challenges faced by women doctors in India, highlighting the legislative, contextual and institutional barriers to wellbeing and advancement for women in the Indian system.
**53.**	Southerst et al.	Diversity of the chiropractic profession in Canada: A cross-sectional survey of Canadian Chiropractic Association members.	17	*Chiropractic & Manual Therapies*	**No**, the concept of profession is not interrogated or explained.	**No**, while the author does make a linkage to the provision of culturally competent healthcare and diversity, equity and inclusion in the profession itself, there is only a descriptive account of the profile of the Canadian medical profession and the extent to which it departs of the rest of the population. It finds that women represent 50% less than their proportion in the population and that there is little racial and ethnic diversity in the profession, with Black and Indigenous people's being under-represented.
**54.**	Staley et al.	Stitching a new garment: Considering the future of the speech-language therapy profession globally.	15	*South African Journal of Communication Disorders*	**No**, the concept of profession is not interrogated or explained.	**Yes**, the construct of profession is explored explicitly in relation to illuminating disparities and inequalities in developed and developing countries in relation to access and terminology within the speech language therapy profession. It problematizes curricula and knowledge for training SLT in developed countries that are largely exported and applied with few modifications. It recognizes that prioritization of knowledge and practice from developed countries may result in services that are neither relevant nor culturally responsive.
**55.**	Taite et al.	Teaching cultural competence in Law School Curricula: An essential step to facilitate diversity, equity, and	16	*Utah Law Review*	**No**, the concept of profession is not interrogated or explained.	**Yes**, sees the establishment of diversity and cultural competency training within the legal profession as imperative towards eliminating bias in the wider justice system as well as inequality within the profession in terms of the representation of disadvantaged groups. It notes that despite the growing diversity in the US the legal profession

		inclusion in the Legal profession.				remains the whitest continuing a historical practice of being an exclusive province of Anglo-Saxon white men.
**56.**	Toraman et al.	The great barrier to teaching profession: Technicism, rethinking the meaning of professionalism through teachers' experience.	26	*International Online Journal of Education and Teaching*	**Yes**, the article discusses SoP literature to discuss what a profession and professionalism is. The fundamental aspect of teaching as a profession is identified as the recognition of the social contribution that person in this occupation render to society.	**No**, the paper identifies two forms of professionalism: teachers as passive technicians and teachers as professional practitioners. It concludes that given the low status of the profession in Turkey although many teachers still identify with the professionalized notion of teaching, many act as technocrats – relaying knowledge but not seeing themselves as active producers and change makers in knowledge production on teaching.
**57.**	Turksoy	The future of public relations, advertising and journalism: How artificial intelligence may transform the communication profession and why society should care?	23	*Turkish review of Communication Studies*	**No**, the construct of profession is not interrogated.	**No**, the sociological literature or approach to profession is not leveraged to understand the impact of AI. To this extent the paper is somewhat inward looking considering the impact of AI on the functions and roles of communication professionals, although some consideration is given to the impact of AI on society at large. It concludes that human critical thinking skills would be the least impacted by AI and that a growing proportion of jobs will require creative intelligence, social intelligence and the ability to leverage AI.
**58.**	Van Spronsen et al.	A new list for choosing wisely Canada from the "hidden profession" of medical laboratory science.	4	*American Journal of Clinical Pathology*	**No**, the construct of profession is not interrogated.	**No**, however there is some discussion on the strengthening of professional identity to facilitate autonomy of decision making in regards to laboratory tests. The paper explores the linkage between the scopes of practice and increasing involvement of medical laboratory professionals and their ability to limit patient harm and reduce wastage that has implication for environmental harm.
**59.**	Varnado et al.	Rejecting the status quo: A call to action using culturally responsive pedagogy in Health Professions Programs.	1	*Journal of Allied Health*	**No**, the construct of profession is not interrogated.	**No**, however recognizes the linkage between the cultural competence of healthcare providers and unequal health outcomes and remaining disparities in healthcare in society. This is also perpetuated by the inequality of access to disadvantaged communities to health professions education. It argues that failure of healthcare providers to build connections with patients may lead to health disparities, preventable differences in the burden of disease, injury, or opportunities for social disadvantaged populations to achieve optimal health.

**60.**	Vinson	Dual loyalty and the medical profession for Australian defence force medical officers.	10	*Journal of Military and Veterans' Health*	**No**. The construct of profession is not interrogated	**No**, the paper is inward looking in so far as it explores the dual loyalty inherent in the practice of a medical professional appointed to the armed/defense force. It outlines the legal frameworks applicable to both roles which gives the medical profession loyalty the primacy, however, within the field complexities arise with balancing these roles (defying orders if instructed not to treat a detained person, or in instances where the individual care has to be juxtaposed against the care of a community).
**61.**	Wagner et al.	Overcoming past perceptions and a profession-wide identity crisis to reflect pharmacy's future.	22	*American Journal of Pharmaceutical Education*	**No**. The construct of profession is not interrogated.	**No**, the paper is in-ward looking in that it attempts to create clearer understandings of the identities and role of the pharmacist alongside changes to the profession and its responsibilities over time and a shift in the educational qualification. The article suggests a tiered system of qualification, similar to nursing to address the different types of identities inherent in pharmacy practice that would allow pharmacists to choose which level of practice and identity suits them.
**62.**	Whelan	On your Marx…? A world to win or the dismantlement of a profession? On why we need a reckoning.	13	*British Journal of Social Work*	**No**. The construct of profession is not interrogated, only attempts to describe what the scope and nature of social work is.	**Yes**, to some extent this paper does this but inadvertently. It questions the claims to social justice as being one of the key principles of the profession by showing the history and emergence of social work within the context of capitalist society as a means of controlling and managing the poor, indigent and less moral groups of society. The paper argues for reclaiming the role of profession as activist and not passivist.
**63.**	Wiggins et al.	Factors that influence scope of practice of the chiropractic profession in Australia: A scoping review.	17	*Chiropractic & Manual Therapies*	**No**. The construct of profession is not interrogated.	**No**. The paper evaluates the factors that influence the scope of practice of the chiropractic profession in Australia. It argues that an established framework has the potential to benefit patient safety, professional identity, public perception, education and regulation of the profession. The review confirmed four factors that influence the scope of practice; education, professional identity, and organizational structure.
**64.**	Wiggins et al.	Factors that influence scope of practice of the five largest health care professions in	14	*Human Resources for health*	**No**. The construct of profession is not interrogated.	**No**. Given recent legislative shifts which changed the responsibility for regulating practice across various health care professions in Australia from profession specific statutes to the National law many professions were unsure of their scopes of practice. The paper identifies eight factors that influence scope of practice: education, competency,

		Australia: A scoping review.				professional identity, role confusion, legislation and regulatory policies, organizational structures, financial factors, and professional and personal factors. It makes the link between clear and defined scopes of practice and the quality of health care delivered to society.
**65.**	Wright	(Re)production of symbolic boundaries between native and non-native teachers in the TESOL profession.	6	*Asian-Pacific Journal of Second and Foreign Language Education*	**No**. The construct of profession is not interrogated.	**Yes**. Focuses on the creation and maintenance of symbolic boundaries, showing the forms of capital that is valued and symbolic violence against outsiders in the English teaching profession. Here native English speakers are symbolized as legitimate while non-native English speakers are not, regardless of levels of qualification to teach English. Draws on Bourdieu to frame the investigation in terms of symbolic violence.
**66.**	Jeong et al.	The advent of the new architectural profession through BIM.	52	*Building*	**No**. The construct of profession is not interrogated.	**No**, professions is not used as a unit of analysis to understand a social problem. Here the focus is inward looking, charting the change in the linkage between the architectural profession and knowledge which has gradually over time become increasingly conceptual. With the advent of knowledge-processing agents the newest architectural professionals seek to process knowledge through the use of algorithm-embedded machines rather than direct conceptualization.

Table 1: Sample for analysis
